# Long-term follow-up of early stage HER2-positive breast cancer patients treated with trastuzumab: A population-based real world multicenter cohort study

**DOI:** 10.3389/fonc.2022.861324

**Published:** 2022-08-02

**Authors:** Sander Ellegård, Kristina Engvall, Mustafa Asowed, Anna-Lotta Hallbeck, Nils Elander, Olle Stål

**Affiliations:** ^1^ Department of Oncology, Linköping University, Linköping, Sweden; ^2^ Department of Biomedical and Clinical Sciences, Linköping University, Linköping, Sweden; ^3^ Department of Oncology, Jönköping, Sweden; ^4^ Department of Clinical Genetics, Linköping University, Linköping, Sweden

**Keywords:** HER2, breast cancer, real-world evidence, adjuvant, trastuzumab (Herceptin), prognostic factors

## Abstract

**Introduction:**

Since its introduction in standard of care, trastuzumab has revolutionized the treatment of patients with early and late stages of HER2-positive breast cancer. While the initial clinical trials were convincing and lead to major changes in practice, more knowledge on the long-term outcome and tolerability is needed. The present study was designed to assess the survival, prognostic factors and relapse patterns after the implementation of trastuzumab in a real-world cohort.

**Methods:**

All cases of HER2-positive breast cancer diagnosed between 2006 and 2014 in the Southeast Healthcare Region of Sweden were retrospectively identified. Medical records were thoroughly reviewed with regard to clinicopathological parameters, treatments, relapse pattern and adverse events.

**Results:**

643 patients were identified and 599 were eligible for analysis. Breast cancer specific survival, distant recurrence free survival and local recurrence free survival were 93.4%, 89.7% and 98.0% for trastuzumab treated patients and 87.4%, 81.6% and 87.4% in patients not treated with trastuzumab, respectively. ER status, nodal status and trastuzumab treatment were all independent prognostic factors in multivariable analysis. No new safety concerns were discovered.

**Conclusion:**

The real-world outcome of trastuzumab-treated patients with early HER2-positive breast cancer is similar to what has been previously reported in long-term follow up of prospective clinical trials. ER status, nodal status and trastuzumab treatment are independent prognostic factors for breast cancer specific mortality rate, distant recurrence rate and locoregional recurrence rate in HER2-positive patients in the trastuzumab era.

## Introduction

Breast cancer is the most common malignancy in women, each year affecting more than two million new individuals around the world (1). Between 15 and 25 percent of all breast cancers are classified as Human epidermal growth factor receptor 2 (HER2) positive (2, 3). Reports of the incidence of HER2-positive disease have varied and 2009, a Swedish study showed a prevalence of 14.3% (4). HER2-is a trans membrane tyrosine kinase receptor without any known ligands, that through homo- and hetero-dimerization with other receptors of the HER family initiate a signaling cascade resulting in tumor cell proliferation and survival (5). HER2 has been identified both as a negative prognostic factor and a treatment predictive target for receptor specific drugs such as the antibody trastuzumab. Since its introduction in standard of care, trastuzumab has significantly improved the outcome of patients with early and late stages of HER2-positive breast cancer (6, 7). Several publications have reported similar outcomes in real-world and randomized controlled trial populations (8, 9). The optimistic long term prognosis for stage I patients has led to de-escalation attempts in terms of the adjuvant systemic therapy offered, including the trial by Tolaney et al. where excellent outcome following paclitaxel weekly in combination with trastuzumab was observed (10). Nevertheless, a substantial proportion of patients with HER2-positive disease will eventually experience locoregional or systemic relapse. In order to potentiate the HER2 targeting treatment, novel drugs and/or combinations of trastuzumab and novel compounds have therefore been explored. Pertuzumab, a monoclonal antibody binding to the HER2 subdomain II, thus inhibiting the heterodimerization of HER2 and other receptors of the HER family, has proven clinically beneficial when combined with trastuzumab (11–13). While the combination of pertuzumab and trastuzumab has been demonstrated to improve the pathological complete response (pCR) rate in the neoadjuvant setting, the role of the combination in adjuvant settings is still under debate (11, 13). On the other hand, the KATHERINE trial demonstrated an additional value of the antibody-drug conjugate compound trastuzumab emtansine for patients with residual disease following neo-adjuvant chemotherapy (14). Furthermore, the tyrosine kinase inhibitor neratinib, which inhibits HER1, HER2 and HER4, were shown to improve long term prognosis for patients with HER2-positive disease, in particular for patients who had estrogen receptor (ER) positive tumors (15).

Despite many treatment options available for patients with HER2-positive breast cancer, there is still a need to learn how to identify patients who benefit from additional treatment. This is particularly important when results from randomized controlled trials are implemented in standard of care, where patients might be different in terms of age, performance status and comorbidities to those who were included in the clinical trials. Real-world follow ups are important in this context, to help us evaluate current treatment strategies and to identify groups of patients where additional studies are necessary.

This study was designed in order to assess the real-world treatment coverage and long-term outcome, including prognostic parameters and recurrence patterns, of patients treated with adjuvant trastuzumab in early stages of HER2-positive breast cancer. All HER2-positive patients since the introduction of adjuvant trastuzumab in the Southeast Health Care Region of Sweden were retrospectively identified and formed the study cohort. The cohort represents a true real-world perspective, as the Scandinavian health care system with public funded free of charge treatment ensures that all citizens regardless of socio-economic status are offered equal care.

## Materials and methods

### Study design and patients

A population based retrospective multicenter cohort study in the Southeast Health Care Region of Sweden was designed. In this region, systemic cancer therapy such as trastuzumab is available at three public funded oncology departments located at the three major hospitals (Linköping, Kalmar, and Jönköping). Approximately 1.1 million citizens live in the region. Patients were identified *via* the national Swedish cancer registry and by using local pathology department databases to ensure the inclusion of all HER2-positive patients.

All female patients diagnosed with HER2-positive breast cancer between 2006-01-01 and 2014-03-13 were included. Exclusion criteria were male sex, stage IV disease, other malignancy affecting treatment and follow up, and incomplete data (e.g. missing medical records). HER2 status was determined according to clinical routine using immunohistochemistry (IHC) and/or fluorescent/chromogenic *in situ* hybridization (FISH/ISH). HER2-positive tumors were defined as either IHC score of 3+ or IHC score of 2+ in combination with confirmed amplification of the HER2 encoding gene with FISH/ISH analysis. A majority of patients received chemotherapy in addition to trastuzumab. The predominant chemotherapy schedule included three cycles of epirubicin, cyclophosphamide and fluorouracil every three weeks followed by either three cycles of docetaxel every three weeks or twelve cycles of weekly paclitaxel. Endocrine therapy was given to ER-positive patients per clinical routine with tamoxifen or aromatase inhibitor with or without GNRH-analogue depending on the menopausal status. ER positivity was defined as per Swedish clinical guidelines as ≥10% positive cells, measured with immunohistochemistry. Local and locoregional radiotherapy were given according to regional- and national guidelines.

Trastuzumab in combination with chemotherapy was initially recommended only for lymph node positive patients. However, this was changed early during the studied interval to the current indication, which includes all patients with tumor size larger than 5 mm with or without lymph node involvement.

Follow-up time was defined as the time from diagnosis until death or loss to follow up. Loss to follow up was defined either as the cutoff date of medical record review or the last date when data of the patient were available in the medical records (e.g. due to emigration).

### Endpoints

Key endpoints were breast cancer-specific survival (BCSS), distant recurrence-free survival (DRFS), and local recurrence-free survival (LRFS). BCSS was defined as survival time from diagnosis until death caused by breast cancer. BCSS rather than overall survival was utilized in order to better reflect trastuzumab’s anti-tumoral effect. This assumption was made due to the retrospective nature of this study where patients not treated with trastuzumab were expected to be older and have more comorbidities, which could lead to an overestimation of the effect of trastuzumab on survival. Censoring was made at loss to follow up or death of other cause. DRFS was defined as the time from diagnosis until first evidence of breast cancer distant metastasis diagnosed either with radiology or cytology/histology. LRFS was defined as the time to local recurrence as diagnosed with biopsy or clinical examination. Secondary endpoints were clinicopathological prognostic factors, safety in terms of adverse events and complications and relapse pattern.

### Statistical analysis

Hazard ratios were calculated using univariate and multivariable Cox regression. P-values <0.05 were considered statistically significant. Survival was calculated using the Kaplan-Meier method and significance was determined using the log-rank test. In the Cox regression analysis age was used as a continuous variable.

Pearsons Chi-square test was used to detect statistical differences in distribution of clinical characteristics between trastuzumab treated and untreated patients.

Fisher’s exact test, using a 2-sided alpha, was used to detect statistical differences between metastatic sites due to the small number of events.

For analysis of hazard ratio and survival related to nodal status, subjects with four to nine positive nodes were combined with those who had more than nine positive nodes due to few patients in both groups. Similarly, and as the frequency of NHG (Nottingham Histological Grade) I tumors was low, patients with NHG I and II were merged and compared with NHG III in all subgroup analyses. SPSS Statistics version 25 (IBM) was used for Cox proportional hazard, Chi-square tests and Fisher’s exact test. The R package in survminer was used to make Kaplan-Meier curves with life tables and calculating log rank statistics.

### Ethical consideration

This study was conducted according to the Helsinki declaration and was approved by the Regional Ethical Review Board in Linköping (original approval DNR M140-06, approved amendments DNR 2014/163-32 and DNR 2020-05501). Based on the retrospective non-interventional design, the Ethical Review Board waived the need for informed consent.

## Results

### Study population and treatment data

**Figure 1:**
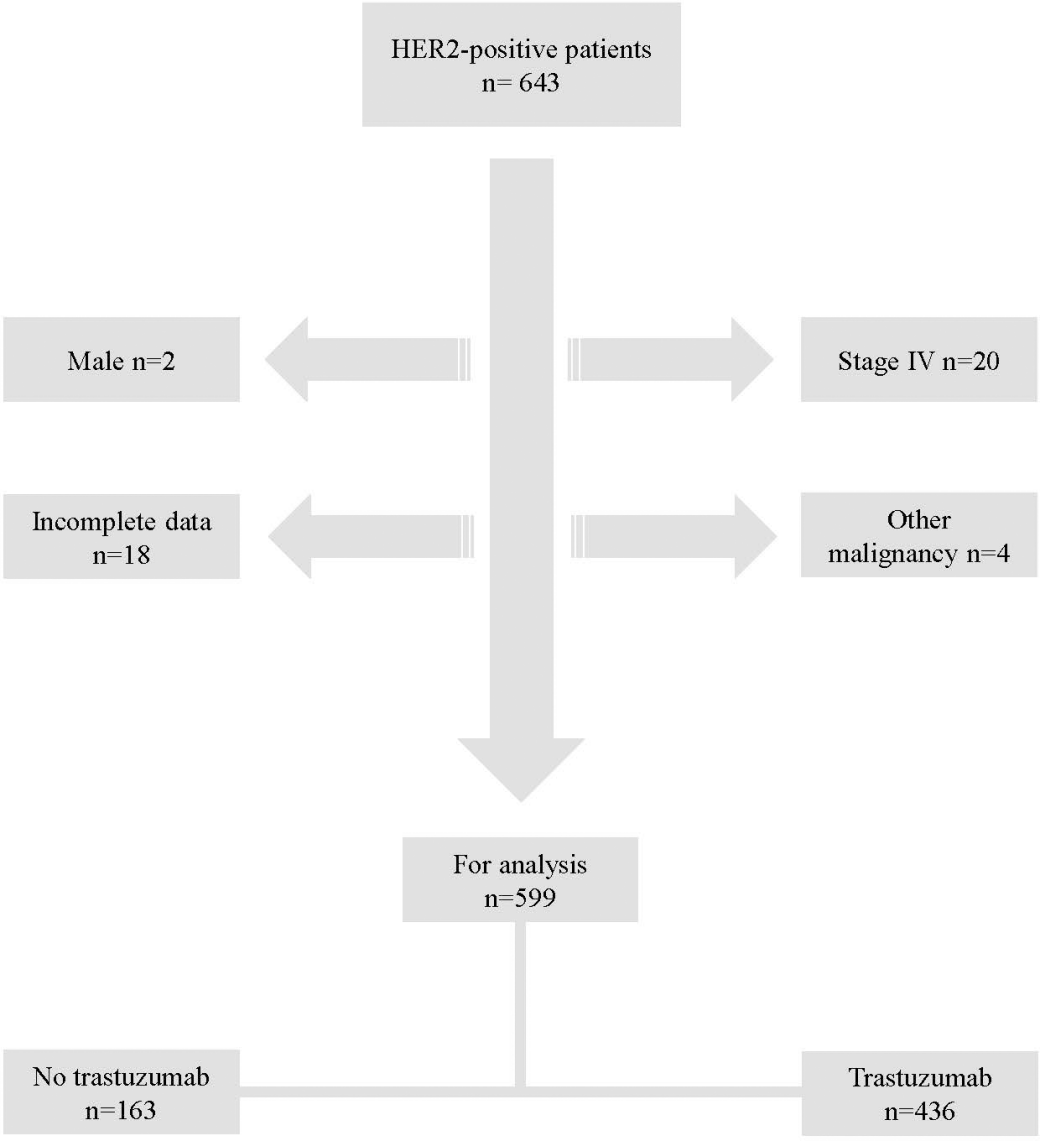
Flow chart of study population.

During the study interval of 2006 – 2014, 643 patients were diagnosed with HER2-positive breast cancer in the Southeast health care region of Sweden. Following the exclusion of 44 patients who did not meet the inclusion criteria, 599 subjects remained and formed the study population (**Figure 1**).

Median follow-up time in the total cohort was 6.8 years (Range: 0.5-13.1 and 95% CI: 6.5-7.1).

The proportion of patients receiving trastuzumab was initially 52-57% in 2006-2008 but increased over time, reaching a maximum of 82-88% in 2011-2013. Patients that did not receive trastuzumab were significantly older, had significantly less nodal involvement, significantly smaller primary tumors, significantly lower tumor grade and did, almost exclusively, not receive chemotherapy (**Table 1**).

**Table 1:** Clinicopathological parameters.

		No trastuzumab	Trastuzumab	
		N (%)	N (%)	p-value
All patients		163 (100)	436 (100)	
Age	<30	1 (1)	5 (1)	**<0.0005**
	30–39	2 (1)	38 (9)	
	40–49	19 (12)	89 (20)	
	50–59	23 (14)	107 (25)	
	60–69	35 (22)	139 (32)	
	70–79	40 (25)	54 (12)	
	80+	43 (26)	4 (1)	
Neoadjuvant treatment	No	157 (96)	385 (88)	**0.003**
	Yes	6 (4)	51 (12)	
Type of surgery	Breast conserving	52 (32)	164 (38)	0.35
	Mastectomy	111 (68)	271 (62)	
Tumor size	<21mm	85 (52)	203 (47)	0.67
	21-50mm	60 (37)	167 (38)	
	>50mm	6 (4)	19 (4)	
	Missing	12 (7)	47 (11)	
ER-status	ER-negative	59 (36)	165 (38)	0.71
	ER-positive	104 (64)	271 (62)	
NHG	NHG I	12 (7)	10 (2)	**0.005**
	NHG II	64 (39)	139 (32)	
	NHG III	86 (53)	261 (60)	
	Missing	1 (1)	26 (6)	
Nodal status	0	111 (68)	184 (42)	**<0.0005**
	1–3	27 (17)	113 (26)	
	≥4	17 (10)	83 (19)	
	Missing	8 (5)	56 (13)	
Histological subtype	Ductal	145 (95)	395 (93)	0.56
	Lobular	3 (2)	16 (4)	
	Other	5 (3)	14 (3)	
Chemotherapy	Yes	26 (16)	420 (96)	**<0.0005**
	No	137 (84)	16 (4)	
Radiotherapy	Yes	81 (51)	348 (80)	**<0.0005**
	No	78 (49)	85 (20)	
	Missing	4	3	

Bold is used when numbers are statistically significant (p<0.05).

During the studied interval, 94% (95% CI: 91.7-96.1%) of the patients that received chemotherapy also received trastuzumab. In 2006, 79.3% of the patients that received chemotherapy were treated with trastuzumab. This proportion increased to 96% in 2009 and then remained above 95% for the rest of the studied interval.

Trastuzumab was prematurely discontinued in 41 (9.4%) of cases. The most common cause for treatment discontinuation was heart toxicity (n=21, 4.8%). Other specified causes that led to discontinuation of trastuzumab were allergic reactions (n=1, 0.2%), pain (n=2, 0.5%), pancreatitis (n=1, 0.2%), infection (n=1, 0.2%) and progressive disease (n=4, 0.9%) although in many cases the cause was not clearly stated (n=11, 2.5%).

### Prognostic factors and events

**Figure 2:**
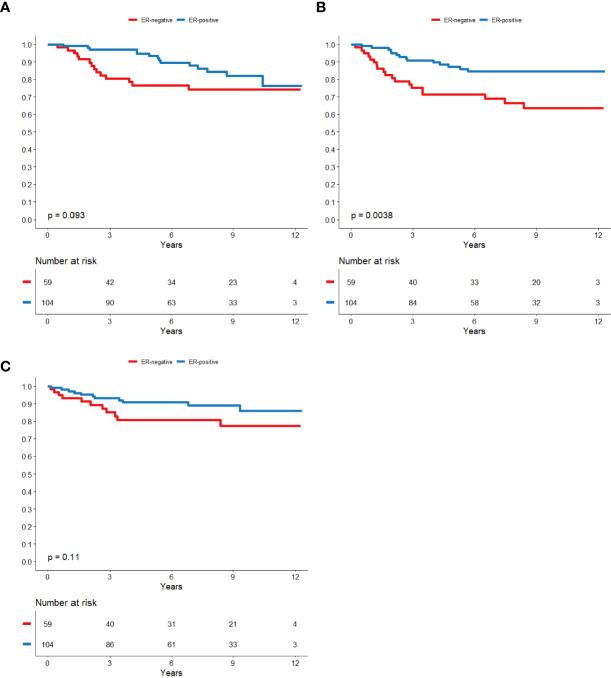
ER-status in relation to BCSS **(A)**, DRFS **(B)** and LRFS **(C)** respectively in patients that did not receive trastuzumab.

**Figure 3:**
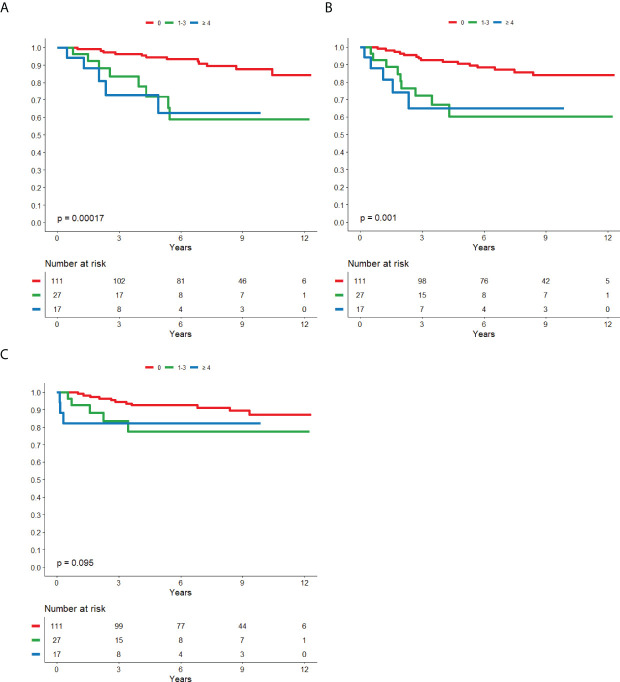
Nodal status in relation to BCSS **(A)**, DRFS **(B)** and LRFS **(C)** respectively in patients that did not receive trastuzumab.

**Figure 4:**
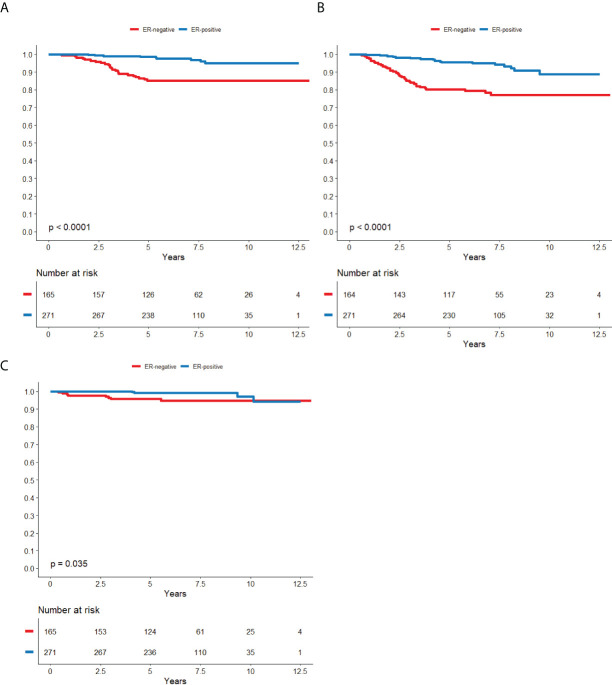
ER-status in relation to BCSS **(A)**, DRFS **(B)** and LRFS **(C)** respectively in patients that was treated with trastuzumab.

**Figure 5:**
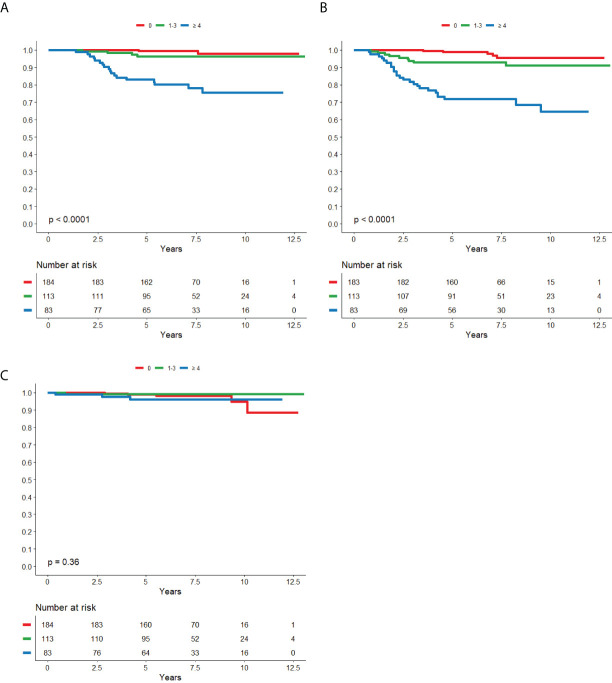
Nodal status in relation to BCSS **(A)**, DRFS **(B)** and LRFS **(C)** respectively in patients that was treated with trastuzumab.

5-year BCSS was 87.4% (95% CI; 82.3-92.0) in the cohort not treated with trastuzumab and 93.4% (95% CI; 91.2-95.6) in the trastuzumab-treated cohort. The 5-year DRFS was 81.6% (95% CI; 76.5-86.7) in the cohort not treated with trastuzumab and 89.7% (95% CI; 87.1-92.3) in the trastuzumab-treated cohort. The 5-year LRFS was 87.4% (95% CI; 82.7-92.0) in the cohort not treated with trastuzumab and 98.0% (95% CI; 96.7-99.3) in the trastuzumab-treated cohort. Kaplan-meier curves of survival with regard to ER and nodal status are displayed in **Figures 2**, **3** for patients not treated with trastuzumab and **Figures 4**, **5** for patients treated with trastuzumab. The five-year BCSS, DRFS and LRFS are shown in **Table 2**.

**Table 2:** Subgroup dependent 5-year survival rates.

	5-year BCSS % (95% CI)		5-year DRFS % (95% CI)		5-year LRFS % (95% CI)	
	No trastuzumab	Trastuzumab	No traztuzumab	Traztuzumab	No traztuzumab	Traztuzumab
ER-positive	84.4 (76.0-92.8)	97.6 (95.6-99.6)	84.3 (76.7-91.9)	95.5 (93.0-98.0)	89.0 (82.3-95.7)	99.2 (98.2-100)
ER negative	74.1 (62.3-85.9)	84.5 (78.8-90.2)	63.4 (49.9-76.9)	80.2 (74.1-86.3)	77.1 (64.8-89.4)	94.7 (91.2-98.2)
Number of metastatic lymph nodes = 0	87.7 (80.6-94.8)	98.3 (96.5-100)	83.9 (76.3-91.5)	98.9 (97.3-100)	89.4 (82.9-95.9)	98.2 (96.2-100)
Number of metastatic lymph nodes = 1-3	58.7 (35.8-81.6)	96.4 (92.9-99.9)	60.0 (38.8-81.2)	92.9 (88.2-97.6)	77.3 (59.3-95.3)	99.1 (97.3-100)
Number of metastatic lymph nodes = >4	62.4 (35.0-89.8))	75.5 (65.1-85.9)	64.9 (39.2-90.6)	71.9 (62.1-81.7)/	82.4 (64.4-100)	96.0 (91.5-100)

Cox proportional hazard analysis revealed that trastuzumab treatment, nodal status and ER-status were significant prognostic factors for breast cancer specific mortality rate (BCSMR), distance recurrance rate (DRR) and local recurrence rate (LRR) when adjusted for age, grade and tumor size (**Table 3**). The adjusted HR for presence vs absence of trastuzumab was improved with regard to all endpoints reflecting the increased rate of unfavorable tumor characteristics in the trastuzumab-treated cohort. Individual Cox proportional hazard models were made for the trastuzumab treated cohort and the cohort not treated with trastuzumab respectively, where nodal status and ER status were the only significant prognostic factors in both cohorts for BCSMR and DRR although not for LRR where no single factor retained its prognostic value (data not shown).

**Table 3:** Multivariable cox regression analysis.

Variable in equation	Univariate HR	Univariate p-value	Multivariable HR	Multivariable p-value
**BREAST CANCER SPECIFIC MORTALITY RATE**				
Trastuzumab	**0.42**	**<0.0005**	**0.19**	**<0.0005**
Nodal status	**2.33**	**<0.0005**	**3.32**	**<0.0005**
ER-status	**0.34**	**<0.0005**	**0.39**	**0.003**
Tumor size	**2.82**	**<0.0005**	1.42	NS
NHG-grade	1.75	0.065	1.85	0.097
Age (continuous)	**1.02**	**0.022**	1.02	NS
**DISTANT RECURRENCE RATE**				
Trastuzumab	**0.56**	**0.008**	**0.31**	**<0.0005**
Nodal status	**2.33**	**<0.0005**	**2.63**	**<0.0005**
ER-status	**0.32**	**<0.0005**	**0.31**	**<0.0005**
Tumor size	**2.74**	**<0.0005**	1.44	0.091
NHG-grade	1.43	NS	1.32	NS
Age (continuous)	1.01	NS	1.01	NS
**LOCOREGIONAL RECURRENCE RATE**				
Trastuzumab	**0.19**	**<0.0005**	**0.13**	**<0.0005**
Nodal status	1.06	NS	**1.65**	**0.048**
ER-status	**0.45**	**0.021**	**0.43**	**0.034**
Tumor size	1.31	NS	1.05	NS
NHG-grade	1.04	NS	1.61	NS
Age (continuous)	1.02	NS	0.98	NS

Bold is used when numbers are statistically significant (p<0.05). NS, Not significant.

### Metastatic sites and metastatic pattern

In total, 87 (15%) patients experienced distant recurrence during the studied interval, 33 of which were in the cohort not treated with trastuzumab and 54 in the trastuzumab treated cohort. The most common metastatic sites were lung (n=41, 47%), liver (n=38, 44%) and bone (n=36, 41%) followed by brain (n=29, 33%) and skin (n=5, 6%).

There were few differences regarding metastatic pattern between the trastuzumab treated and untreated subgroups. Overall, 29 (4.8% (95% CI; 3.3-6.8)) patients were diagnosed with brain metastasis during the studied interval, with no significant difference between trastuzumab treated and untreated patients. These 29 cases corresponded to 33.3% (95% CI; 24.1-43.7) of all patients who experienced distant metastasis, still with no significant difference between trastuzumab treated and untreated subjects (39% (95% CI 27-52) vs. 28% (95% CI 12-40). However, at the time of the first distant recurrence, brain metastases were significantly more common in patients treated with trastuzumab (n=14, 26% (95% CI 16-50)) as compared to the patients not treated with trastuzumab (n=2, 6% (95% CI 1-18)), p=0.023. No other significant differences in metastatic pattern were observed (**Table 4**).

**Table 4:** Metastatic site for patients with distant recurrences.

		No trastuzumab	Trastuzumab	
Metastatic site		N (%)	N (%)	p-value
Lymph node	No	20 (61)	41 (76)	NS
	Yes	13 (39)	13 (24)	
Cutaneous	No	31 (94)	51 (94)	NS
	Yes	2 (6)	3 (6)	
Bone	No	16 (49)	35 (65)	NS
	Yes	17 (52)	19 (35)	
Lung	No	17 (52)	29 (54)	NS
	Yes	16 (49)	25 (46)	
Liver	No	19 (58)	30 (56)	NS
	Yes	14 (42)	24 (44)	
Brain	No	25 (76)	33 (61)	NS
	Yes	8 (24)	21 (39)	
Brain metastasis as first distant recurrence	No	31 (94)	40 (74)	**0.023**
	Yes	2 (6)	14 (26)	
Other metastatic site	No	23 (70)	45 (83)	NS
	Yes	10 (30)	9 (17)	

Bold is used when numbers are statistically significant (p<0.05). NS, Not significant.

Forty six of 50 (92%) biopsy confirmed recurrent/metastatic lesions (all locations combined) were HER2-positive. HER2-negative recurrence occurred in three trastuzumab-treated patients and in one patient not treated with trastuzumab.

## Discussion

This population-based cohort study describes the long-term outcome and relapse pattern in a real-world cohort covering all patients with early stages of HER2-positive breast cancer, both those treated with trastuzumab in the adjuvant setting and those who did not undergo such treatment, under a period of eight years. The results demonstrate that the long-term prognosis for patients treated with trastuzumab is similar to what has been seen in early clinical trials (16) as well as more recent studies with similarly distributed patients such as the PERSEPHONE trial (17). The main prognostic factors for breast cancer survival, distant recurrence and locoregional recurrence were lymph node status, ER-status, and trastuzumab treatment.

The proportion of patients with early stage HER2-positive breast cancer undergoing adjuvant treatment with trastuzumab was relatively low during the first years after the introduction, only reaching 57% in 2006, which is somewhat lower than corresponding data from the Netherlands (18) but slightly higher than 50% that was reported from New Zeeland and Australia (19). The proportion of patients receiving trastuzumab increased over time, to a maximum of 88% (2011) and then remained over 80% for the rest of the studied interval in this study. The optimal percentage is difficult to determine due to the fact that some patients with HER2-positive disease will not actually benefit from the treatment or be suitable for it due to their health status in general or various contraindications. It is likely to believe that part of this change was attributable to changes in national and international practice guidelines (20, 21), and to some extent it might also reflect accumulated knowledge and clinical experience. In addition, the prescription of adjuvant trastuzumab is closely linked to the prescription of adjuvant chemotherapy, the latter now being recommended and offered to a larger proportion of patients due to more data regarding chemotherapy in elderly patients (22). Notably, since 2009 more than 95% of the patients undergoing adjuvant chemotherapy also underwent trastuzumab treatment in parallel which is in line with current Swedish national guidelines.

In accordance with early trials on the topic, brain metastases were notably common as first metastatic lesion in patients experiencing relapse under follow up (6). In the present cohort, 25% of the patients who experienced relapse after trastuzumab treatment presented with brain metastases, which is in the upper range of what was previously reported (23). However, brain metastases were common regardless of trastuzumab treatment and overall brain recurrences did not significantly differ between trastuzumab treated and untreated patients. The high prevalence of brain metastases in patients with metastatic disease is in line with previous data from our group (24). These results further strengthen the current theory that the increased number of brain metastases noted in early trastuzumab trials is due to trastuzumab´s proportionally higher effect on extracranial disease. We do believe that these data suggest that metastatic screening of the brain is warranted at the time of distant recurrence for patients with HER2-positive breast cancer. Furthermore, the incidence of local recurrences in patients treated with trastuzumab was low (n=12, 3%) which is noteworthy as HER2-positive breast cancer was associated with an increased risk of local recurrence in the pre-trastuzumab era (25). These data are in line with more recent publications regarding trastuzumab-treated patients where local recurrences are reported in 2-4% of the patients (26, 27).

Adverse events attributed to trastuzumab were rare, and we could not identify any new safety concerns with regard to trastuzumab. Heart toxicity is one of the most common adverse events that lead to discontinuation, however only 4.9% discontinued trastuzumab treatment due to heart toxicity in our study, a low figure compared to early trials and other studies (6, 28). The low occurrence of heart toxicity could be due to the use of epirubicin instead of doxorubicin or improved clinical awareness and understanding of how to handle trastuzumab´s heart toxicity over time. The retrospective nature and real-world focus of the present study could result in an underestimation of less serious adverse events, not leading to treatment discontinuation, as data collection exclusively relied on what was evident from the medical records and no standardized CTCAE scorings or similar were available.

The main strengths of the present study include the truly real-world perspective, as all HER2-positive patients in a large geographical region diagnosed under a period of eight years, regardless socioeconomic status, performance status, and comorbidity, were included. The long follow up ensures that mature survival data, rather than surrogate endpoints, were available.

Self-evidently, long term follow-up of patients who received trastuzumab and other anti-HER2 targeting treatments in the last few years is not yet possible, and the results here presented do not necessarily fully reflect the predicted outcome of today’s state of the art treatment strategies. Another limitation is that important prognostic variables such as node status and tumor size could not reliably be extracted from the medical records for the neoadjuvant patients in this study, hence extrapolation to this growing subgroup of patients should be done with caution. Due to the retrospective nature of the study, no additional examinations or sampling have been made and no reliable comorbidity score was deemed possible to calculate from the medical records. Importantly, trastuzumab and chemotherapy were almost exclusively administered together in this study, hence we could not evaluate the survival benefit by trastuzumab as compared to chemotherapy alone.

In conclusion, adjuvant treatment with trastuzumab is a tolerable and effective treatment when prescribed according to current clinical guidelines and praxis and confer a favorable prognosis for patients with HER2-postive breast cancer. However, using prognostic variables identified in this study patients with unfavorable prognosis can be identified, e.g., patients with ER negative disease and four or more nodal metastases treated with trastuzumab only had a 65.6% (95% CI; 54.8-76.4) 5-year BCSS, a survival rate much lower than for the overall study-population emphasizing the need for increased efforts to improve the treatment for these patients.

## Conclusion

This study provides real world evidence supporting the data from early clinical trials regarding the excellent long-term outcome of adjuvant trastuzumab in patients with early HER2-positive breast cancer. ER status, nodal status and trastuzumab treatment were the only individual factors significantly associated with the long-term prognosis in this cohort. Among the minority of patients who experienced distant recurrence brain metastases were common and brain metastasis diagnosed as first metastatic relapse was more common in patients treated with trastuzumab.

## Data availability statement

The raw data supporting the conclusions of this article will be made available by the authors, without undue reservation.

## Ethics statement

The studies involving human participants were reviewed and approved by Regional Ethical Review Board in Linköping. Written informed consent for participation was not required for this study in accordance with the national legislation and the institutional requirements.

## Author contributions

SE, KE, A-LH, NE, and OS designed the study. SE, KE, and MA collected and organized the data. SE and OS analyzed the data. All authors interpreted and discussed the data, were major contributors to the manuscript, and read and approved the final manuscript.

## Funding

This study was supported by grants from the Swedish Cancer Society (grant number 17-0479), Medical research council of South-Eastern Sweden (FORSS, grant number RÖ-966444), ALF Grants Region Östergötland (Grant number LIO-795201) and Stiftelsen Onkologiska Klinikernas Forskningsfond i Linköping (2018–12–19).

## Conflict of interest

The authors declare that the research was conducted in the absence of any commercial or financial relationships that could be construed as a potential conflict of interest.

## Publisher’s note

All claims expressed in this article are solely those of the authors and do not necessarily represent those of their affiliated organizations, or those of the publisher, the editors and the reviewers. Any product that may be evaluated in this article, or claim that may be made by its manufacturer, is not guaranteed or endorsed by the publisher.
